# Full body mobile brain-body imaging data during unconstrained locomotion on stairs, ramps, and level ground

**DOI:** 10.1038/sdata.2018.133

**Published:** 2018-07-10

**Authors:** Justin A. Brantley, Trieu Phat Luu, Sho Nakagome, Fangshi Zhu, Jose L. Contreras-Vidal

**Affiliations:** 1 Laboratory for Non-Invasive Brain Machine Interfaces, Department of Electrical & Computer Engineering, University of Houston, Houston, TX 77056, USA

**Keywords:** Neural circuits, Motor control, Electroencephalography - EEG, Electromyography - EMG

## Abstract

Human locomotion is a complex process that requires the integration of central and peripheral nervous signalling. Understanding the brain’s involvement in locomotion is challenging and is traditionally investigated during locomotor imagination or observation. However, stationary imaging methods lack the ability to infer information about the peripheral and central signalling during actual task execution. In this report, we present a dataset containing simultaneously recorded electroencephalography (EEG), lower-limb electromyography (EMG), and full body motion capture recorded from ten able-bodied individuals. The subjects completed an average of twenty trials on an experimental gait course containing level-ground, ramps, and stairs. We recorded 60-channel EEG from the scalp and 4-channel EOG from the face and temples. Surface EMG was recorded from six muscle sites bilaterally on the thigh and shank. The motion capture system consisted of seventeen wireless IMUs, allowing for unconstrained ambulation in the experimental space. In this report, we present the rationale for collecting these data, a detailed explanation of the experimental setup, and a brief validation of the data quality.

## Background & Summary

Human bipedal locomotion results from the complex interaction of dynamic networks in the spinal cord with feedback mechanisms originating from sensory inputs through spinal and supraspinal pathways. Fundamental circuitry in the spinal cord are believed to be responsible for generating basic locomotor patterns, while descending pathways from the brain are thought to provide signalling that can initiate, terminate, and guide locomotion^1^. Traditionally, many studies on locomotion have relied on invasive experiments in the spinal cord and motor cortices of cats^1^. However, invasive electrophysiological approaches are not feasible to study the involvement of cortical networks in human walking due to the ethical challenges of invasive recordings in human subjects; thus, the role of the spinal cord and brain during human bipedal locomotion has been investigated using non-invasive imaging modalities^2–4^. Many studies using functional magnetic resonance imaging (fMRI) and magnetoencephalography (MEG)—both stationary methods—have relied on gait imagery or observation^2–4^ as a source for understanding cognition and gait. While motor imagery and observation may result in the activation of networks involved in actual task execution^5–12^, stationary studies do not allow for the investigation of networks connecting cortical brain activity and muscular activation involved in the action. Thus, we must rely on mobile systems for simultaneous collection of information from the central and peripheral nervous systems.

Electroencephalography (EEG) is a powerful tool for studying the brain’s involvement in movement execution with a high temporal resolution. On the down side, EEG lacks the spatial resolution of other techniques and suffers from a significantly attenuated signal due to volume conduction^13,14^. The localization of the EEG signal sources in the brain can be challenging, since the volume conduction model is comprised of multiple layers, including fluid media and anisotropic tissues^13,14^. Fortunately, there are methods for extracting highly localized information, even with spatial resolution, and the high temporal resolution provides detailed information related to the task. These characteristics make EEG a suitable source for brain imaging during an overground locomotion task.

EEG has been used to investigate cortical involvement during steady-state walking^15–26^ and multi-tasking^27^. However, most of these studies have been limited to treadmills, which fix the cadence and speed of locomotion. In this report, we present a highly comprehensive dataset from ten healthy individuals during a multi-terrain walking task. The subjects were instrumented with non-invasive EEG, EMG, and full body motion capture while walking on a custom gait course including level ground, ramps, and stairs. To the best of our knowledge, this dataset is the first recording of continuous overground walking while the subjects were instrumented with EEG. Furthermore, the addition of muscle and kinematic information enhances the richness of this unique dataset. A major concern of EEG is the presence of physiological and non-physiological artifacts during movement. Previous studies have shown that with a careful setup, motion artifacts are negligible at lower walking speeds^28^.

Previous datasets have been published with EEG data during seated cognitive tasks^29,30^, during a stimulus-viewing task after transcranial magnetic stimulation (TMS)^31^, and during perceptual tasks during simultaneous EEG, fMRI, and MEG^32^. Luciw *et al*.^33^ published a comprehensive dataset containing EEG, EMG, kinematic, and kinetic data from twelve health subjects during a grasp and lift. To our knowledge, this is the first publicly available dataset that includes full brain and body imaging (EEG, EMG, kinematics) during overgound multi-terrain locomotion.

We have previously used this dataset to investigate corticomuscular coherence^34^, cortical activations during multi-terrain walking^35,36^, and the neural dynamics associated with transitions between walking terrains^37^. Additionally, we have used this dataset to demonstrate the ability to continuously predict muscle activation patterns directly from EEG^34,38^ and joint angles from EMG signals^39^. The multi-modal nature of this data sets enables future studies in brain-computer interfaces (BCIs) and motor control during human locomotion, such as:

- Investigation of corticomuscular coherence in the source domain
- Analysis of motion artifacts in EEG during multi-terrain walking
- Design of an offline decoder for continuous prediction of joint angles from EEG
- Biomechanical analysis of gait and muscle activation patterns during multi-terrain walking (e.g., symmetry, stride length)

## Methods

### Participants

Ten healthy individuals (five males, five females; ages 18-31 years old) with no history of neurological disorder, lower-limb pathology, or gait abnormalities participated in this study. The experimental protocol and informed consent (reviewed and signed by each participant) were approved by the Institutional Review Board (IRB) at the University of Houston. All experiments were performed in accordance with the 45 Code of Federal Regulations (CFR) part 46 (“The Common Rule”), specifically addressing the protection of human study subjects as promulgated by the U.S. Department of Health and Human Services (DHHS).

### Instrumentation & Data Collection

**Figure 1:**
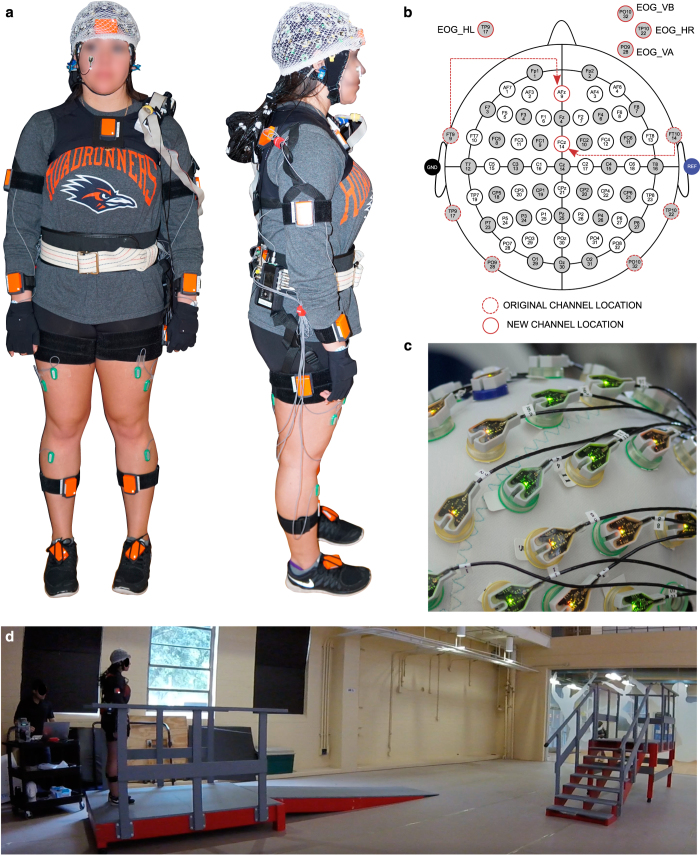
Fully instrumented subject and experimental gait course. (**a**) Able-bodied subject instrumented with EEG, EMG, and full-body motion capture. (**b**) EEG channel montage and EOG locations. EOG_HL: horizontal left, EOG_HR: horizontal right, EOG_VA: vertical above, EOG_VB: vertical below. (**c**) Close-up image of active EEG electrodes. (**d**) Experimental gait course including level walking, ramps, and stairs.

The participants were instrumented with a non-invasive EEG cap, EMG, and full body motion capture for comprehensive brain-body imaging during an over ground multi-terrain locomotion task. Figure 1 shows a fully instrumented subject.

Each subject’s head circumference was measured to allow for selection of an appropriately sized EEG. A 64-channel Ag/AgCl active electrode EEG setup (BrainAmp DC and MOVE, Brain Products GmbH, Germany) was used to wirelessly record from the face and scalp (sampling frequency=1000 Hz). The data were recorded using the BrainVision Recorder software (Brain Products GmbH, Germany). Channels TP9, PO9, PO10, and TP10 were removed from the cap and used for electrooculography (EOG) to capture blinks and eye movements. The remaining 60 channels were arranged according to the modified international 10–20 system with slight modifications. Electrodes at channel locations FT9 and FT10 were moved to locations AFz and FCz, respectively, and the ground and reference were placed on the earlobes (A1 and A2). This modification was made to provide additional coverage over the frontal cortex. The EOG channels were arranged such that PO9 and PO10 were positioned superior and inferior to the right eye, respectively, while TP9 and TP10 were placed on the left and right temples on the sides of the face, respectively.

In preparation for the experiments, subjects were asked to refrain from using products in their hair that may increase the impedance at the scalp/electrode interface (e.g., conditioner, hair gel, etc.). Prior to donning the cap, the skin on the face around the eyes, the temples, and the earlobes were gently cleaned with alcohol wipes to remove any dirt and skin oils. The cap was aligned on the head such that the FP1 and FP2 were 10% of the distance from the nasion to the inion along the midsaggital plane, and electrode Cz as at the vertex of the head. After donning the cap, a conductive electrolyte gel was applied between the electrode tips and the scalp to reduce the interface impedance. The impedance was maintained below 50 kΩ and in most cases reduced to below 20 kΩ. The channel impedances were recorded prior to the start of the experiment. After completing the gelling process, reference markers were placed on the nasion and left/right pre-auricular points to identify anatomical landmarks. A 3D scanning system (BrainVision Captrak, Brain Products GmbH, Germany) was used to record the 3D electrode positions on the scalp. Finally, the reference markers were removed, and a light surgical mesh was placed over electrodes to fix their location on the scalp and to mitigate the potential influence of motion artifacts during the walking tasks.

Surface EMG signals were recorded using active bipolar electrodes (fixed electrode distance of 20 mm) at 1000 Hz (SX230 sensors and DataLOG MWX8, Biometrics Ltd, Newport, UK). The data was stored on an onboard micro SD card and simultaneously streamed to a PC for visualization and data monitoring using the Biometrics Analysis Software (Biometrics Ltd, Newport, UK). Surface EMG was recorded from six sites bilaterally: tibialis anterior, gastrocnemius, rectus femoris, vastus lateralis, bicep femoris longus, and the semitendinosus. The individual sensor locations were estimated based on limb measurements and anatomical landmarks and validated using recommended clinical tests (i.e., asking subjects to perform a movement and palpating the muscle cite to identify a contraction)^40^. The recording sites were shaved to remove any hair and subsequently cleaned with an alcohol solution and allowed to dry. The sensors were then fixed to the skin using a double-sided adhesive tape (designed for specific sensor as not to obstruct electrodes). The signals were checked for quality and sensor locations were adjusted as necessary. In cases where the signal was persistently noisy, even after skin preparation, an abrasive skin preparation gel was applied to the cite to remove dead cells and reduce the impedance. The signals were referenced to an electrode located on the skin at the bony prominence created by the styloid process of the ulna. The muscle recording sites were identified published guidelines.

The subjects were instrumented with 17 wireless inertial measurement units (Xsens MVN, Xsens North America Inc., Culver City, CA) for full body motion capture. Each unit was positioned on the body according the manufacturer’s instructions, which included the following locations on the body: (1) head, (2) sternum, (3,4) L/R scapula, (5,6) L/R mid-upper arm, (7,8) L/R mid-forearm, (9,10) L/R hand, (11) L5 spine, (12,13) L/R mid-thigh, (14,15) L/R mid-shank, and (16,17) L/R foot. After donning the 17 IMUs, the system was calibrated while the subject stood in a neutral posture with their hands by their side. The subject was then asked to move about the experimental space, and the accuracy of the movements was validated by visual inspection. Additionally, the magnetic field was continuously monitored to ensure minimal interference and data corruption. The data were recorded at either 30 or 60 Hz (indicated in data) using the MVN Studio software (version 4.4, Xsens North America Inc., Culver City, CA). Information regarding the coordinate systems and algorithms implemented by the software can be found in Roetenberg *et al.*^41^

The data were time synchronized using a custom hardware trigger and aligned using MATLAB R2016a (The Mathworks Inc., Natick, MA).

### Experimental Protocol

The experiments were conducted on a custom-built gait course (shown in Fig. 1) incorporating five steady locomotion modes: level ground walking (LW), stair descent (SD), stair ascent (SA), ramp descent (RD), and ramp ascent (RA). Prior to beginning the walking trials, the subjects were asked to stand quietly with their eyes open faced away from the gait course (facing a blank wall). One minute of EEG and EMG were recorded to establish a baseline period of brain and muscle activity. Motion capture was not recorded during this period. For the walking trials, the subjects were asked to walk at their preferred speed and to minimize excessive head movements, eye blinks, and jaw movements (e.g., talking, clenching). The subjects began level walking, descended the ramp, gradually turned and transitioned to level ground walking, ascended the staircase, and came to a stop at the end of the stair platform (forward direction). The subject would complete a 180° turn, descend the stairs, transition to level ground, ascend the ramp, and come to rest (reverse direction). Ambulation in both the forward and reverse direction of the course was considered one complete test trial (Fig. 1). The subjects were asked to complete the walking trials in ten blocks of two trials. This was done to allow subjects time to rest between trials if needed and to minimize any potential data loss due to hardware malfunction. The subjects completed an average of twenty trials in approximately ten blocks.

## Data Records

All published data are de-identified and subjects gave written informed consent for their data to be openly shared on a credible public data repository. All data files are available from FigShare (Data Citation 1) and have been made available under the terms of Attribution 4.0 International Creative Commons License (http://creativecommons.org/licenses/by/4.0/). The data are archived in a single file set and organized with the following naming convention:

$${AB}_{-}{UH}_{-}xx-Tyy-abc.ext,$$

where *xx* is the subject number (01,02,…,10), *yy* is the trial number (00, 01, 02,…), *abc* is the data type (eeg, emg, kin, chanlocs, impedance),*.ext* is the file format (.mat,.bvct), and the bold text is fixed for all files. It should be noted that the *chanlocs*, *impedance,* and *emgsensitvity* data are recorded once at the beginning of the session, so they will not contain the **-T***yy* entry in the file name. The trials start at *00* indicating the rest, or baseline, trial; all trials thereafter (01, 02, 03,…) are locomotion trials.

### -chanlocs.bvct

The -chanlocs.bvct is an XML format file that contains the digitized EEG electrodes locations in 3D space. The data were collected using the BrainVision Captrak software (Brain Products). The file includes the EEG cap size (circumference of the head in cm), the subject’s head shape (round or oval), and the Cartesian/polar coordinates for each electrode (x, y, z, θ, φ, r).

### -impedance.mat

The -impedance.mat file contains a structure with two fields (described in Table 1; variable name: *impedance*).

### -eeg.mat

The EEG data are stored in a structure containing two fields (described in Table 2; variable name: *eeg*). The data stored in *eeg.rawdata* are raw and unfiltered data without the four EOG channels, which have been moved to eeg.eogdata. In addition to the raw data, the processed data used in ref. 36 have been provided. The data were first high-pass filtered using a fourth-order zero-phase Butterworth filter at 0.01 Hz. Next, channels with a standard deviation greater than 1000 μV or a kurtosis more than five standard deviations from the mean kurtosis of all channels were rejected (using *pop_rejchan.m* from the EEGLAB toolbox^42^ for MATLAB). The retained channels were referenced to the common average. Next, we applied artifact subspace reconstruction (ASR)^43^ on a sliding window of 500 ms to remove corrupted sections with high amplitude bursts (burst criterion=3). Finally, Infomax independent component analysis (ICA) was applied to decompose data into independent sources. Only the ICA sphering and weight matrices are provided in the dataset to reduce size, however, the ICA activations can be easily computed as *ICA weights× ICA sphering matrix × processed data.* It is important for anyone using these datasets to consider the processing pipeline in detail, as EEG processing can be highly application specific. Otherwise, the raw data should be used to process the data accordingly.

### -emg.mat

The EMG data are stored in a structure with two fields (described in Table 3; variable name: *emg*), each corresponding to a specific leg (*emg.left, emg.right*).

The channels for both correspond to the following muscles (in the order of the rows from top to bottom): tibialis anterior (TA), medial gastrocnemius (GAST), vastus lateralis (VL), rectus femoris (RF), semitendinosus (SEM), biceps femoris longus (BFL).

### -emgsensitivity.mat

The -emgsensitivity.mat file contains a structure with two fields (described in Table 4; variable name: *emgsensitivity*).

### -kin.mat

The -kin.mat file contains a structure (described in Table 5 (available online only); variable name: *kin*) with the full body motion capture data. The structure contains theee fields, *.srate*, *.setup* and *.data*, with the latter two containing numerous subfields. Table 5 (available online only) breaks down the information contained in each of the fields and subfields.

## Technical Validation

### Data synchronization

**Figure 2:**
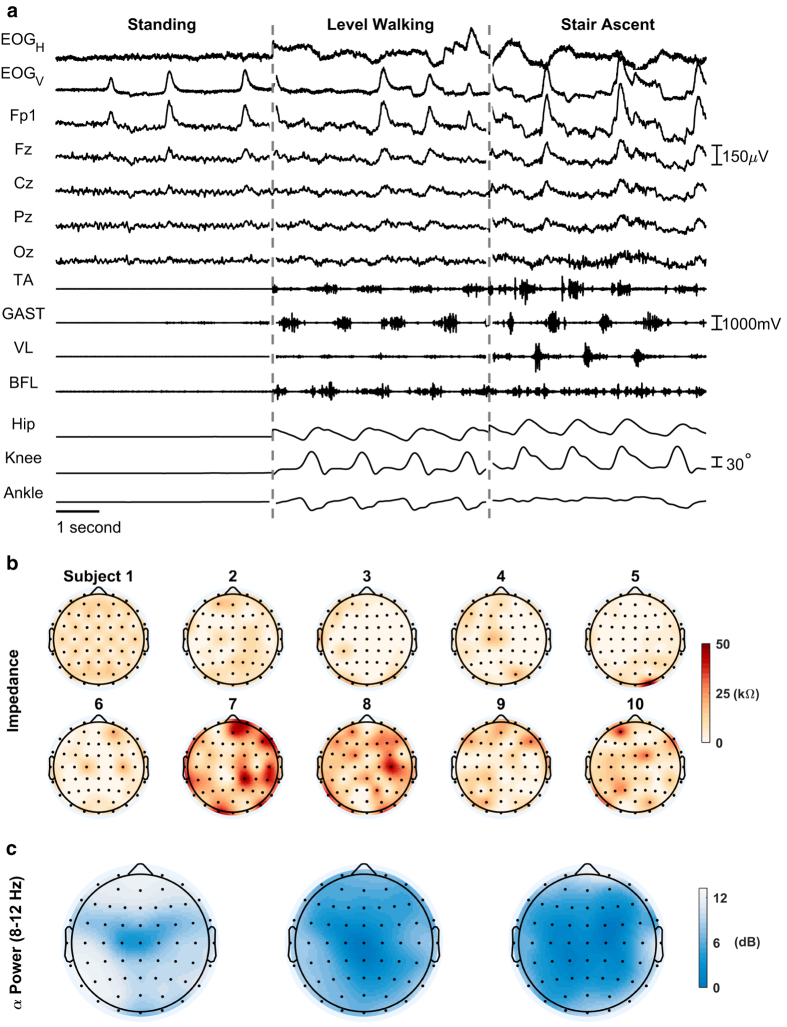
Time-synchronized subset of EOG, EEG, EMG, and kinematic data; channel impedances (kΩ) of the 60-channel EEG; and terrain-specific α power (dB) in all the EEG channels. (**a**) The timeseries EOG_H_ (horizontal) and EOG_V_ (vertical) are computed as bipolar signals for the horizontal and vertical EOG channels, respectively. (**b**) Impedance values (kΩ) of the 60-channel EEG for each of the ten subjects just prior to beginning the experiment. (**c**) The total α power (dB) in all the EEG channels (60 electrodes) during each of the conditions (standing, LW, SA, from left to right) is shown above the timeseries plot. Gradual increases in α band desynchronization can be observed between as the subject transitions from standing, to level ground walking, to stair walking, respectively.

This multi-modal dataset contains recordings from multiple devices/software; thus, external hardware was required for proper synchronization. The hardware consisted of a push-button connected to a 5 V power source. Each system was connected via its respective cable connector. Digital pulses were placed within the data at the beginning and end of each trial and aligned in post processing. The provided data are aligned but in otherwise raw form. This allows future users to process the raw data as desired (e.g., filtering). An example subset of the time-synchronized data is shown in Fig. 2a.

### EEG, Captrak, and Impedance data

The EEG setup was rigorously prepared to minimize potential artifacts and improve the signal-to-noise ratio (complete details of this are included in the methods). The impedance for each electrode was reduced to below 50 kΩ prior to beginning the experiment (shown in Fig. 2b).

Event related desynchronization (reduction in power or ERD) in the α and β band during movement onset has been widely documented in the literature^44,45^. This characteristic behavior is observed in our data. As shown in Fig. 2, there is a clear reduction in α power when the subject is walking on level ground when compared to standing. Furthermore, there is an additional reduction in α power during stair ascent when compared to level ground walking. The α power (dB) scalp maps shown in Fig. 2c were computed using the following steps: (1) band pass filtered between 0.3–50 Hz using a fourth order Butterworth filter; (2) referenced to the common average (using *reref.m* in MATLAB); (3) the power spectral density estimate was computed for each channel using the Thomson multitaper method (*pmtm.m* in MATLAB; 3.5 discrete prolate spheroidal sequences); and the average log power in the α band (8–12 Hz) for each channel was projected onto the scalp using the *topoplot.m* function in the EEGLAB toolbox for MATLAB^42^.

### EMG data

The quality of the lower-limb EMG signals was assessed prior to the beginning of the experiment. The gains were adjusted so that none of the channels were subject to digital clipping. As shown in Fig. 2a, the muscle activations can be clearly seen during the walking conditions (LW and SA). Furthermore, the activations of antagonistic muscles (e.g., TA/GAST and VL/BFL) are approximately 180° out of phase as expected during locomotor tasks.

### Kinematic data

**Figure 3:**
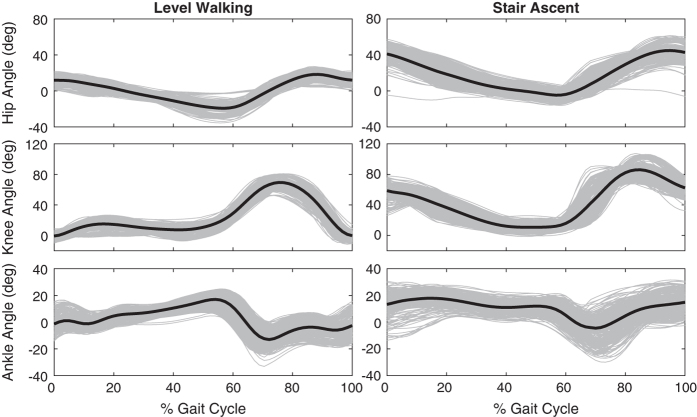
Lower limb joint angles from the right leg of all ten subjects and all trials during level walking and stair ascent. Each grey line represents the joint trajectory during a single gait cycle and the black line represents the mean.

The motion capture system was carefully setup according to the manufacturer’s instructions. After donning the 17 IMUs, the system was calibrated while the subject stood in a neutral posture with their hands by their side. The subject was then asked to move about the experimental space, and the accuracy of the movements was validated by visual inspection. Finally, the magnetic field was continuously monitored to ensure minimal interference and data corruption. Figure 3 shows the hip, knee, and ankle joint angles for all ten subjects during locomotion on level ground (left column) and stairs (right column).

## Additional information

**How to cite this article**: Brantley, J. A. *et al*. Full body mobile brain-body imaging data during unconstrained locomotion on stairs, ramps, and level ground. *Sci. Data* 5:180133 doi: 10.1038/sdata.2018.133 (2018).

**Publisher’s note**: Springer Nature remains neutral with regard to jurisdictional claims in published maps and institutional affiliations.

## Supplementary Material



## Figures and Tables

**Table 1:** Contents of *impedance* structure in -impedance.mat files.

impedance.channel	Channel names, including 60-channel EEG, 4-channel EOG, ground, and reference.
impedance.value	Impedance values in kΩ

**Table 2:** Contents of *eeg* structure in -eeg.mat files.

eeg.data	*60*×*N* matrix containing the EEG data. The rows are the channels corresponding to the channel locations in the corresponding -captrack.bvht and the columns are the *N* sample points. (units: μV)
eeg.eogdata	*4*×*N* matrix containing the EOG data, where the rows correspond to the following channels (shown in Fig. 1): (1) horizontal left temple (EOG_HL), (2) horizontal right temple (EOG_HR), (3) vertical above (EOG_VA), and (4) vertical below (EOG_VB). (units: μV)
eeg.rejectedchannels	Vector containing indices of rejected channels (e.g., 1, 7, 32,..)
eeg.processdata	*(60 – Number rejected channels)*×*N* matrix containing the processed EEG data. The rows are the channels retained for analysis and the columns are the *N* sample points. (units: μV)
eeg.icasphere	*(60 – Number rejected channels)*×*(60 – Number rejected channels)* matrix containing ICA sphering matrix
eeg.icaweights	*(60 – Number rejected channels)*×*(60 – Number rejected channels)* matrix containing ICA weight matrix

**Table 3:** Contents of *emg* structure in -emg.mat files.

emg.left	*6*×*N* matrix containing data for left leg, where the rows are the channels and the columns are the *N* sample points (units: mV)
emg.right	*6*×*N* matrix containing data for right leg, where the rows are the channels and the columns are the *N* sample points (units: mV)

**Table 4:** Contents of *emgsensitivity* structure in -emgsensitivity.mat files.

emgsensitivity.channel	EMG channel names corresponding to channel abbreviations above. The labels are prefixed by either an “L_” for left leg or an “R_” for right leg (e.g., R_TA for right tibialis anterior)
emgsensitivity.value	Channel sensitivity values in V

**Table 5:** Contents of *kin* structure in -kin.mat files

kin.setup	.segmentlabel	*23*×*1* cell array containing the names of the body segments	
	.sensorlabel	*17*×*1* cell array containing the sensor placement sites	
	.jointlabel	*22*×*1* cell array containing the joint names	
	.numTrials	Scalar value indicating the number of times the gait course was completed for that session (down and back indicates one completion). This is either one or two.	
kin.data	.sensorAcceleration	.Pelvis.T8.Head.RightShoulder.RightUpperArm.RightForeArm.RightHand.LeftShoulder.LeftUpperArm.LeftForeArm.LeftHand.RightUpperLeg.RightLowerLeg.RightFoot.LeftUpperLeg.LeftLowerLeg.LeftFoot	N×3 matrix containing sensor acceleration vector (x,y,z) at each time point N (units: m/s^2^)
	.sensorAngularVelocity		N×3 matrix containing sensor angular velocity vector (x,y,z) at each time point N (units: rad/s^2^)
	.sensorOrientation		N×3 matrix containing sensor orientation vector (x,y,z) at each time point N in the global frame (units: m)
	.sensorMagneticField		N×3 matrix containing sensor magnetic field vector (x,y,z) at each time point N (units: arbitrary)
	.orientationQuaternion	.Pelvis.L5.L3.T12.T8.Neck.Head.RightShoulder.RightUpperArm.RightForeArm.RightHand.LeftShoulder.LeftUpperArm.LeftForeArm.LeftHand.RightUpperLeg.RightLowerLeg.RightFoot.RightToe.LeftUpperLeg.LeftLowerLeg.LeftFoot.LeftToe	N×4 matrix containing segment orientation quaternion (q0, q1, q2, q3) at each time point N
	.orientationEuler		N×3 matrix containing segment orientation (x,y,z) at each time point N (units: m)
	.position		N×3 matrix containing the position vector (x, y, z) of the origin of the segment in the global frame at each time point N (units: m)
	.velocity		N×3 matrix containing the velocity vector (x, y, z) of the origin of the segment in the global frame at each time point N (units: m/s)
	.acceleration		N×3 matrix containing the acceleration vector (x, y, z) of the origin of the segment in the global frame at each time point N (units: m/s^2^)
	.angularVelocity		N×3 matrix containing the angular velocity vector (x, y, z) of the segment in the global frame in (units: rad/s)
	.angularAcceleration		N×3 matrix containing the angular acceleration vector (x, y, z) of the segment in the global frame in (units: rad/s^2^)
	.jointAngle	.jL5S1.jL4L3.jL1T12.jT9T8.jT1C7.jC1Head.jRightC7Shoulder.jRightShoulder.jRightElbow.jRightWrist.jLeftC7Shoulder.jLeftShoulder.jLeftElbow.jLeftWrist.jRightHip.jRightKnee.jRightAnkle.jRightBallFoot.jLeftHip.jLeftKnee.jLeftAnkle.jLeftBallFoot	N×3 matrix containing the Euler representation of the joint angle vector (x, y, z) calculated using the Euler sequence ZXY (units: deg)
	.centerOfMass	N×3 matrix containing the body Center of Mass (x,y,z) in the global frame at each time point N (units: m)	
kin.srate	Scalar value indicating sampling rate of system (30 Hz or 60 Hz)		
